# The prevalence of malnutrition and growth percentiles for urban South African children

**DOI:** 10.1186/s12889-019-6794-1

**Published:** 2019-05-02

**Authors:** Lukhanyo H. Nyati, John M. Pettifor, Shane A. Norris

**Affiliations:** MRC/Wits Developmental Pathways for Health Research Unit, Department of Paediatrics and Child Health, Faculty of Health Sciences, University of the Witwatersrand, 7 York Rd, Parktown, Johannesburg, 2193 South Africa

**Keywords:** Stunting, Wasting, Overweight & obesity, Growth references, South Africa

## Abstract

**Background:**

Low- and middle-income countries (LMIC) are experiencing a double-burden of malnutrition characterised by high prevalence of both under- and over-nutrition. We set out using data from the mixed-longitudinal Birth-to-Twenty Plus (Bt20+) birth cohort, to evaluate the patterns of malnutrition and growth in a large South African (SA) city by; (i) assessing the prevalence of undernutrition from birth to 5 years of age and overweight and obesity from ages 2 to 21 years in black and white, male and female children, and (ii) determining percentiles for height, weight, BMI, waist and hip circumferences and comparing the centiles to American and Dutch references.

**Methods:**

Height, weight, waist and hip circumferences were measured on urban black and white SA children from the Bt20+. A total of 3273 children born between April and June 1990 in the Greater Johannesburg Metropolitan area were included in the cohort. Z-scores were derived using the WHO 2006 child growth standards (0–5 years), for defining stunting, underweight and wasting. The International Obesity Task Force (IOTF) references were used to define overweight and obesity. Percentiles were developed using the lambda mu sigma (LMS) method and compared to American and Dutch references.

**Results:**

Black children were consistently shorter and black males lighter than white children and American references. The prevalence of stunting peaked at 2 years and was significantly higher in males than females and in black than white children. Black females had a greater prevalence of overweight and obesity than black males from 10 to 17 years. The percentiles for black females for weight and BMI were similar to those of South African white and American references but both black and white South African females had lower waist circumferences than American references.

**Conclusion:**

The growth percentiles show that young South African urban black females are experiencing general but not central obesity due to a secular change which is faster in weight than height. High levels of undernutrition persist alongside high levels of over-nutrition with adolescence being a critical period for the upsurge in obesity in females. Early intervention is needed to combat the rise in obesity.

**Electronic supplementary material:**

The online version of this article (10.1186/s12889-019-6794-1) contains supplementary material, which is available to authorized users.

## Background

Low-and middle income countries (LMIC) are experiencing a double-burden of malnutrition with an upsurge in overweight and obesity developing alongside persistently high levels of stunting [1]. Globally, a three-fold increase in the prevalence of obesity has been observed since 1975, while in Africa, there has been a 50% increase in overweight in children under 5 years of age [2]. The World Health Organisation (WHO) estimates that in 2016 18% of children aged 5 to 19 years globally were overweight or obese [3]. Notwithstanding the rise in overweight and obesity, the prevalence of stunting remains persistently high, at about 40% in Africa, a level that has remained stagnant since 1990 and shows little prospect of improvement [4]. The longitudinal assessment of growth and the social, economic, nutritional, behavioural and health environment in which it takes place can help identify critical periods associated with a rise in the prevalence of malnutrition and factors contributing to its development.

Bourne et al. showed that since 1940, South Africa has been undergoing a nutrition transition with an increase in the contribution of fats and a decrease in the contribution of carbohydrates and fibre towards energy consumption [5]. The transition is faster in the black than other populations [5] and in urban that rural populations [6], driving an upsurge in obesity in the black population. Historically, black children and adolescents of both sexes were shorter and lighter [7, 8], and gained adipose tissue at a slower rate than white children [9]. Previously, urban children from average socio-economic status (SES) communities were shorter and lighter than those in rural and well-off urban communities suggesting that a move to an urban environment without an improvement in SES does not benefit growth [10].

When compared with children from a high income country (HIC), South African children were historically shorter, lighter and had lower adipose tissue [11, 12]. More recently, data from the mixed-longitudinal Bt20+ cohort have shown that while South African urban children remain shorter and lighter than the National Centre for Health Statistics (NCHS) references, weight-for-height is greater or similar between birth and 5 years of age [13]. Thus, while South African children continue to falter in linear growth, there is an indication of an early increase in adiposity. There is a need to study these patterns through childhood and adolescence and the mixed-longitudinal data from the Bt20+ cohort provides an opportunity to examine black and white urban children and to assess the changing patterns in obesity.

The current increase in obesity could be as a result of a general increase in obesity throughout the population or due to an increase within a subgroup of the population [14]. Generating percentiles can demonstrate shifts in the centile lines over time to indicate whether the positive secular change in BMI is influenced by those in the upper centiles becoming heavier or an upward shift in all centiles. There are no percentiles for height, weight and BMI for South African children and thus it is not possible to assess the change in patterns that contribute to the secular trends in BMI. Thus, the aims of this study in urban South African children were; (i) to assess the prevalence of undernutrition from birth to 5 years of age and overweight and obesity from ages 2 to 21 years in black and white, male and female children, and (ii) to determine percentiles for height, weight, BMI, waist and hip circumferences and comparing the centiles to American and Dutch references.

## Methods

Data for this study were obtained from the Birth to Twenty Plus study (Bt20+), a prospective longitudinal cohort of children born in the Greater Johannesburg region, South Africa. The majority of the black participants lived in Soweto, a “township” (underdeveloped suburban area) on the outskirts of the greater Johannesburg municipality, housing an estimated 40% of the close to 4 million people living in the city [15, 16]. Soweto was created in the early 1930s by the government which formalised the separation of residential areas for black and white citizens under the system of apartheid. The onset of this study coincided with the political changes leading to the end of apartheid.

Details of the cohort are described elsewhere but are briefly summarised here [17–19]. The study enrolled children born during the 7 weeks [18] between April 23 and June 81,990. Mothers with a permanent residential address in the municipal region were asked to participate in the study. Mothers were recruited at different time points, initially at antenatal clinics, then at delivery, and at 3 months, 6 months, and 1 year post-delivery [17]. A total of 3273 mothers (black 78%, coloured 12%, Indian 4% & white 6%) with singleton births who met the selection criterion of continued residence in the area for 6 months were enrolled into the study [18]. Due to the low number of white children in the original cohort, an additional number of white children born in the same period in 1990 were recruited at age 9 years. The government classification of population groups based on “race” was used (black for African, coloured for those of mixed ancestry, Indian for those whose forebears had immigrated from the Indian subcontinent and white for European-descent) and was assigned based on the parents’ self-reported racial classification. The black population in the Soweto-Johannesburg area is composed of different tribal groups identified by self-reported home language. Genetic variation among black participants had been previously assessed and showed that the cohort is largely homogeneous with a small contribution from diverse assortment of ancestral populations [20].

### Anthropometric measurements

Weight was measured from birth while height was measured from 3 months to 24 years of age in black and white children. Waist and hip circumferences were measured from 9 to 24 years. Birth weight was obtained from health records; no data was available for birth length. Trained fieldworkers measured weight and length at 3, 6 and 12 months. From age 1 year, anthropometric measurements were collected on calendar year data collection cycles using standard methods [21, 22]. Between the birth and 8 years, height was measured using the method of Cameron [21], while from 10 years onwards all anthropometric measurements were done using the method of Lohman et al. [22]. The methods are similar and both advocate stretch height to reduce diurnal variation. All measurements up to data collection year 8 were done during home visits, while later measurements were done at the research centre. Before age 2 years, supine length was measured from the vertex of the head to the bottom of the heels, with the child lying flat on its back, head positioned in Frankfort vertical plane and knees straightened. After age 2 years, height was measured without shoes to the nearest 0.1 cm using a Harpenden Stadiometer (Holtain, U.K.). Weight was measured on an electronic scale to the nearest 0.1 kg. Waist circumference was measured half way between the iliac crest and lowest rib at the end of normal expiration to the nearest 0.1 cm with no clothing barrier. Hip circumferences was measured over the greater trochanter and the most protruding part of the buttocks to the nearest 0.1 cm with minimal clothing. Children with very low birth weight (< 1500 g) were excluded from the estimation of undernutrition, given the likelihood of them having had a complicated postnatal course with respiratory, gastrointestinal and neurological complications, possibly leading to impaired growth during childhood. All participants (over the age of 11 years) and their guardians provided written informed assent and consent respectively and ethics approval was obtained from the University of the Witwatersrand Committee for Research on Human Subjects (M010556).

### Generating growth percentiles

Growth percentiles were generated using the LMS function within the generalized additive models for location scale and shape (gamlss version 5.0–1) package in R [23, 24]. The LMS method summarizes the distribution of the data by age and sex in terms of three curves called lambda (L), mu (M) and sigma (S) [23]. The M curve is the median by age, the S curve is the coefficient of variation, and the L curve expresses the skewness of the distribution in terms of the Box-Cox power needed to transform the data to near normality. Plots for the Bt20+ percentiles for height, weight and BMI were superimposed on plots for CDC growth references [25], while plots for the Bt20+ waist circumference references were superimposed on plots from the NHANES III [26]. For height, weight and BMI, the 3rd, 50th and 97th centile are plotted while for waist circumference there being no centile lines below the 10th centile in the NHANES III data, the 10th, 50th and 90th centiles are presented. Plots for the Bt20+ hip circumference references were superimposed on Dutch references [27]. Data for the Dutch references were available for the − 2, 0 and + 2 standard deviation scores (SDS) corresponding to the 2.3rd, 50th and 97.7th centiles [27].

### Generating Z-scores & BMI categories

The World Health Organization (WHO) 2006 child growth standards for children between birth and 5 years were used to develop height-for-age (HAZ), weight-for-age (WAZ), BMI-for-age (BMIZ) and weight-for-height (WHZ) Z-scores [28]. Stunting, underweight and wasting were defined as a standard deviation score (SDS) < − 2 of the median HAZ, WAZ and WHZ respectively. Overweight and obesity were defined using the International Obesity Task Force (IOTF) age-specific cut-offs for children aged 2 to 216 months [29]. For children older than 216 months the adult cut-offs of BMI > = 25 & < 30 kg/m^2 and BMI > = 30 kg/m^2 were used to define overweight and obesity respectively.

### Statistical analysis

Data were stratified into yearly age groups, which were defined as incorporating all children whose integer age was the same as the age group. For example, age group 1 included all children who had reached 1 year but were less than 2 years. Exceptions to the above definition were age group ‘less than 1’, which only included data for infants aged 3 months to less than 1 year and age group ‘21’, which included all participants aged 21 years and above. Data were analysed using R version 3.3.1 and Stata version 13.1 (Stata-Corp LP, College Station, Texas, USA). Z-scores were generated using the WHO 2006 and WHO 2007 R macros [30, 31]. The programme automatically generates variables, flagging biologically implausible values. These latter values were excluded from the analyses. Additional cleaning to remove outliers was achieved using the plotclean function in the SITAR package (version 1.0.9) in R [32].

Race and sex differences in the proportions of stunting, underweight, wasting, overweight and obesity were assessed using a Pearson Chi-squared test. Race differences in height, weight and BMI were assessed using an independent t-test. All tests were performed at the 95% confidence interval.

## Results

### Race differences in height, weight, BMI, waist and hip circumference and waist-to-height ratio

The number of participants in each age group of the Bt20+ are presented in Table 1a. As the cohort represented the demographics of children in Soweto-Johannesburg, South Africa, a higher number of black than white children were enrolled in the study. Cross-sectional means of height, weight, BMI, waist and hip circumferences and waist-to-height ratio are presented in Table 1. With the exception of age less than 1 year and mid-childhood (5 and 8 years of age), white children were consistently taller than black children from early childhood to young adulthood. The earliest significant race differences were observed at 1 year in males and at 2 years in females such that the mean difference increased from 3.7 cm at 1 year to 7.9 cm at 19 years in males (*p* < 0.0001) while in females the mean difference changed from 4.3 cm at 2 years to 5.2 cm at 19 years (*p* < 0.0001).

**Table 1:** Race differences in (a) height/length, (b) weight, (c) BMI, (d) waist circumference, (e) hip circumference, and (f) waist-to-height ratio (%) from birth to adulthood. Sample sizes are given in Table 1(a) in square brackets. Length was not measured at birth

(a) Height						
Age Group	Males Mean + SD (cm) [N]			Females Mean + SD (cm) [N]		
	Black	White	*P*-value	Black	White	*P*-value
Birth	NA [1245]	NA [152]	NA	NA [1324]	NA [148]	NA
< 1	67.8 (6.9) [417]	67.1 (7.0) [66]	0.39	66.6 (6.8) [423]	66.2 (6.9) [48]	0.708
1	77.0 (4.7) [376]	80.7 (4.7) [23]	**0.001**	75.8 (4.7) [422]	77.8 (5.2) [24]	0.085
2	83.4 (3.6) [296]	87.8 (4.8) [31]	**< 0.0001**	82.6 (3.8) [300]	86.9 (3.9) [24]	**< 0.0001**
5	100.5 (4.0) [519]	104.5 (6.2) [18]	0.382	100.4 (4.3) [560]	102.5 (2.8) [17]	0.22
8	125.4 (6.1) [372]	125.1 (6.7) [15]	0.881	124.6 (5.9) [407]	126.1 (4.5) [12]	0.283
9	132.1 (5.9) [251]	137.1 (5.9) [42]	**< 0.0001**	132.8 (5.6) [253]	136.0 (6.6) [39]	**0.005**
10	137.4 (6.2) [182]	143.3 (7.2) [73]	**< 0.0001**	139.2 (6.3) [159]	142.9 (7.5) [65]	**0.001**
11	142.8 (6.7) [470]	148.7 (7.7) [73]	**< 0.0001**	145.8 (7.4) [505]	148.3 (8.0) [69]	**0.018**
12	147.6 (7.5) [603]	155.8 (9.0) [72]	**< 0.0001**	151.4 (6.8) [685]	155.0 (7.9) [78]	**< 0.0001**
13	153.8 (8.3) [613]	164.0 (9.9) [57]	**< 0.0001**	155.5 (6.2) [690]	160.3 (6.7) [63]	**< 0.0001**
14	160.2 (8.4) [701]	169.8 (9.1) [58]	**< 0.0001**	157.3 (5.9) [754]	162.9 (6.8) [74]	**< 0.0001**
15	165.6 (7.8) [759]	174.8 (8.4) [58]	**< 0.0001**	158.4 (6.2) [841]	163.8 (6.9) [61]	**< 0.0001**
16	168.6 (7.1) [685]	176.6 (7.8) [53]	**< 0.0001**	158.8 (5.9) [724]	164.5 (6.6) [61]	**< 0.0001**
17	170.3 (6.7) [502]	177.7 (8.8) [48]	**< 0.0001**	159.3 (6.2) [561]	165.3 (6.8) [61]	**< 0.0001**
18	171.2 (6.6) [449]	178.0 (8.0) [37]	**< 0.0001**	159.4 (6.1) [427]	166.3 (7.1) [36]	**< 0.0001**
19	170.0 (6.8) [55]	177.9 (7.7) [40]	**< 0.0001**	158.9 (6.5) [47]	164.1 (6.9) [42]	**< 0.0001**
20	171.3 (6.9) [103]	174.3 (6.8) [12]	0.183	158.8 (5.9) [99]	164.5 (6.7) [7]	0.066
21	171.6 (6.6) [695]	178.4 (7.4) [17]	**0.002**	159.7 (6.1) [711]	166.2 (7.2) [23]	**< 0.0001**
(b) Weight						
Age Group	Males Mean + SD (kg)			Females Mean + SD (kg)		
	Black	White	*P*-value	Black	White	*P*-value
Birth	3.1 (0.5)	3.3 (0.5)	**0.019**	3.0 (0.5)	3.1 (0.5)	0.098
< 1	8.3 (1.9)	7.6 (2.0)	0.083	7.8 (1.7)	7.3 (2.0)	0.098
1	10.5 (1.7)	11.0 (1.7)	0.158	10.1 (1.7)	10.2 (2.0)	0.738
2	11.5 (1.7)	13.0 (1.6)	**< 0.0001**	11.4 (1.6)	12.0 (1.4)	**0.037**
5	16.0 (2.0)	16.5 (2.9)	0.814	15.8 (2.1)	16.4 (0.7)	0.188
8	25.2 (3.9)	28.1 (9.2)	0.228	24.9 (4.7)	26.1 (3.7)	0.312
9	28.8 (5.0)	32.7 (7.4)	**0.023**	29.6 (6.4)	30.3 (6.3)	0.541
10	32.6 (6.6)	35.6 (6.2)	**0.009**	34.7 (8.3)	36.1 (8.1)	0.248
11	35.7 (8.0)	39.8 (7.3)	**< 0.0001**	38.9 (10.0)	40.5 (9.4)	0.189
12	39.5 (8.6)	45.6 (10.3)	**< 0.0001**	44.8 (10.9)	46.4 (11.0)	0.244
13	44.1 (9.9)	53.2 (12.1)	**< 0.0001**	50.1 (11.9)	51.8 (10.9)	0.228
14	49.2 (10.6)	59.4 (12.8)	**< 0.0001**	53.2 (11.5)	55.5 (10.9)	0.094
15	53.8 (10.4)	64.9 (12.6)	**< 0.0001**	55.7 (11.7)	57.0 (10.1)	0.343
16	56.9 (9.9)	66.7 (10.8)	**< 0.0001**	57.6 (12.1)	59.2 (10.6)	0.276
17	59.0 (9.8)	71.3 (12.2)	**< 0.0001**	59.0 (11.5)	60.1 (12.3)	0.494
18	59.8 (9.7)	71.1 (12.9)	**< 0.0001**	59.1 (11.9)	63.1 (12.9)	0.079
19	63.1 (14.5)	73.9 (16.5)	**0.013**	61.4 (13.8)	61.9 (15.2)	0.881
20	62.5 (11.0)	68.6 (8.1)	**0.033**	60.4 (12.8)	69.8 (9.4)	**0.039**
21	63.6 (11.6)	75.1 (10.7)	**0.004**	65.1 (16.1)	59.9 (10.2)	**0.027**
(c) BMI						
Age Group	Males Mean + SD (kg/m^2)			Females Mean + SD (kg/m^2)		
	Black	White	*P*-value	Black	White	*P*-value
Birth	NA	NA	NA	NA	NA	NA
< 1	17.9 (2.3)	16.7 (1.6)	**< 0.0001**	17.5 (2.0)	16.2 (1.9)	**0.001**
1	17.7 (1.8)	16.9 (1.7)	**0.044**	17.5 (1.9)	16.7 (1.6)	**0.041**
2	16.5 (2.2)	16.9 (1.6)	0.197	16.7 (2.0)	15.9 (1.8)	0.067
5	15.8 (1.3)	15.0 (0.9)	0.259	15.6 (1.3)	15.6 (0.6)	0.990
8	15.9 (1.5)	17.7 (4.5)	0.146	16.0 (2.1)	16.4 (2.0)	0.509
9	16.4 (2.0)	17.3 (3.1)	0.105	16.7 (2.8)	16.2 (2.1)	0.224
10	17.2 (2.6)	17.3 (2.1)	0.875	17.8 (3.4)	17.5 (2.6)	0.522
11	17.4 (3.1)	18.0 (2.5)	0.099	18.2 (3.8)	18.3 (2.9)	0.866
12	18.0 (2.9)	18.7 (3.3)	0.111	19.4 (4.0)	19.1 (3.2)	0.366
13	18.5 (3.2)	19.6 (3.2)	**0.018**	20.6 (4.4)	20.0 (3.4)	0.186
14	19.1 (3.3)	20.5 (3.5)	**0.039**	21.4 (4.3)	20.8 (3.6)	0.179
15	19.6 (3.1)	21.2 (3.4)	**0.009**	22.2 (4.4)	21.2 (3.4)	**0.042**
16	20.0 (3.0)	21.3 (2.7)	**0.013**	22.8 (4.5)	21.8 (3.3)	**0.027**
17	20.3 (3.0)	22.5 (3.1)	**< 0.0001**	23.2 (4.4)	21.9 (4.0)	**0.019**
18	20.4 (2.8)	22.4 (3.6)	**0.017**	23.2 (4.4)	22.8 (3.9)	0.490
19	21.8 (5.1)	23.3 (5.0)	0.168	24.3 (5.0)	22.9 (4.8)	0.178
20	21.3 (3.3)	22.6 (2.8)	0.146	23.9 (4.7)	25.8 (2.7)	0.142
21	21.6 (3.7)	23.5 (2.4)	0.055	25.5 (6.1)	21.7 (3.9)	**0.001**
(d) Waist circumference						
Age group	Males Mean + SD (cm)			Females Mean + SD (cm)		
	Black	White	*P*-value	Black	White	*P*-value
9	57.1 (4.9)	59.9 (8.1)	**0.046**	57.2 (6.5)	57.7 (5.9)	0.648
10	58.7 (5.8)	60.8 (5.6)	**0.008**	58.4 (6.9)	59.5 (7.0)	0.277
11	60.6 (6.8)	63.4 (6.6)	**0.001**	61.4 (7.5)	63.0 (8.7)	0.167
12	62.6 (6.9)	66.1 (7.2)	**< 0.0001**	63.8 (7.8)	65.3 (8.4)	0.154
13	64.7 (7.5)	69.3 (7.8)	**< 0.0001**	67.3 (9.0)	67.7 (7.8)	0.709
14	67.0 (7.6)	72.1 (8.8)	**< 0.0001**	69.3 (9.1)	69.1 (8.5)	0.814
15	69.1 (7.8)	73.7 (9.6)	**< 0.0001**	71.0 (9.6)	69.6 (8.7)	0.233
16	69.4 (6.5)	75.6 (7.1)	**< 0.0001**	71.9 (9.8)	73.7 (9.4)	0.146
17	71.3 (7.2)	77.7 (7.4)	**< 0.0001**	74.3 (9.8)	74.8 (10.4)	0.701
18	72.6 (7.0)	77.1 (5.4)	**0.002**	76.0 (11.3)	78.2 (11.7)	0.396
19	73.3 (11.3)	81.7 (14.6)	**0.021**	72.7 (10.2)	67.9 (6.4)	**0.022**
20	71.7 (7.6)	76.7 (6.0)	**0.020**	69.3 (8.2)	76.4 (8.8)	0.080
21	75.7 (9.1)	82.9 (6.8)	**< 0.0001**	81.1 (13.2)	72.4 (9.3)	**< 0.0001**
(e) Hip circumference						
Age group	Males Mean + SD (cm)			Females Mean + SD (cm)		
	Black	White	*P*-value	Black	White	*P*-value
9	63.4 (6.2)	66.3 (8.2)	**0.044**	65.8 (9.3)	66.3 (7.4)	0.746
10	71.0 (6.8)	73.7 (6.6)	**0.004**	75.1 (8.9)	75.6 (7.5)	0.670
11	76.4 (8.0)	78.0 (6.7)	0.088	80.3 (9.6)	79.0 (7.8)	0.201
12	78.6 (8.1)	81.5 (7.9)	**0.004**	85.2 (10.2)	84.6 (9.4)	0.635
13	81.5 (8.9)	86.2 (8.6)	**< 0.0001**	90.9 (10.4)	90.4 (8.9)	0.673
14	84.4 (8.4)	89.8 (8.6)	**< 0.0001**	94.3 (10.0)	92.6 (8.1)	0.079
15	87.5 (8.4)	92.5 (9.7)	**< 0.0001**	95.7 (10.0)	94.4 (7.2)	0.193
16	89.3 (7.4)	92.8 (7.2)	**0.001**	96.8 (9.6)	95.5 (8.5)	0.221
17	90.8 (7.8)	94.0 (7.0)	**0.002**	99.5 (9.5)	95.2 (8.3)	**< 0.0001**
18	90.8 (6.9)	92.5 (5.6)	0.196	99.4 (10.2)	97.6 (8.2)	0.339
19	90.7 (10.5)	97.1 (11.0)	**0.027**	100.0 (11.9)	93.8 (8.4)	**0.020**
20	90.8 (7.6)	95.0 (6.9)	0.075	98.6 (10.9)	101.1 (6.4)	0.378
21	93.3 (8.5)	99.1 (6.0)	**0.001**	104.6 (12.9)	96.1 (8.8)	**< 0.0001**
(f) Waist-to-height ratio						
Age group	MalesMean + SD			Females Mean + SD		
	Black	White	*P*-value	Black	White	*P*-value
9	43.0 (3.3)	43.6 (5.3)	0.514	42.8 (4.0)	42.3 (3.3)	0.458
10	42.7 (3.8)	42.4 (3.2)	0.510	42.0 (4.3)	41.6 (4.0)	0.596
11	42.4 (4.2)	42.8 (4.4)	0.508	42.1 (4.6)	42.4 (4.8)	0.601
12	42.4 (4.0)	42.4 (4.2)	0.912	42.1 (4.9)	42.1 (4.8)	0.888
13	42.1 (4.6)	42.3 (4.2)	0.777	43.3 (5.7)	42.2 (4.3)	0.057
14	41.8 (4.7)	42.5 (5.0)	0.327	44.0 (5.7)	42.6 (5.1)	**0.021**
15	41.7 (4.7)	41.8 (4.2)	0.843	44.8 (6.0)	42.6 (5.3)	**0.003**
16	41.2 (3.8)	42.8 (3.6)	**0.002**	45.3 (6.3)	44.7 (5.3)	0.418
17	41.8 (4.2)	43.8 (3.8)	**0.001**	46.7 (6.3)	45.4 (6.2)	0.097
18	42.4 (3.9)	43.6 (3.0)	0.082	47.6 (6.9)	47.5 (6.3)	0.935
19	43.2 (7.0)	46.2 (8.4)	0.150	45.8 (6.4)	41.3 (3.6)	**0.001**
20	41.9 (4.4)	44.1 (4.1)	0.110	43.7 (5.2)	46.4 (4.6)	0.181
21	44.2 (5.3)	46.5 (3.2)	**0.009**	50.8 (8.3)	43.6 (5.6)	**< 0.0001**

Age group < 1 = age between 3 months and 1 year; 21 = age 21 years or greater

*p*-values less than 0.05 are in bold

Similarly for weight, white males, who were 200 g heavier at birth (*p* = 0.019), were consistently heavier than black males except in infancy and mid-childhood. Mean differences increased from 2.5 kg (*p* < 0.001) to 10.8 kg (*p* = 0.013) between ages 2 and 19 years. In females, there were no consistent differences in weight between black and white participants.

BMI was greater in black than white infants during the first 2 years of life. The reverse was observed in males during adolescence (white males having significantly greater BMI than black males between 13 and 18 years). Black females had significantly greater BMI than white females from ages 15 to 17 years.

White males had greater waist and hip circumferences than black males at most ages except at 11, 18 and 19 years for hip circumference. Waist-to-height ratios were similar in black and white males except at ages 16, 17 and 21 years when white males had greater waist-to-height ratio than black males. In females, there were no race differences in waist and hip circumferences except at ages 19 and 21 years for waist circumference, and at ages 17, 19 and 21 years for hip circumference. Black females had greater waist-to-height ratio than white females at ages 14, 15, 19 and 21 years.

### Patterns of malnutrition

**Fig. 1:**
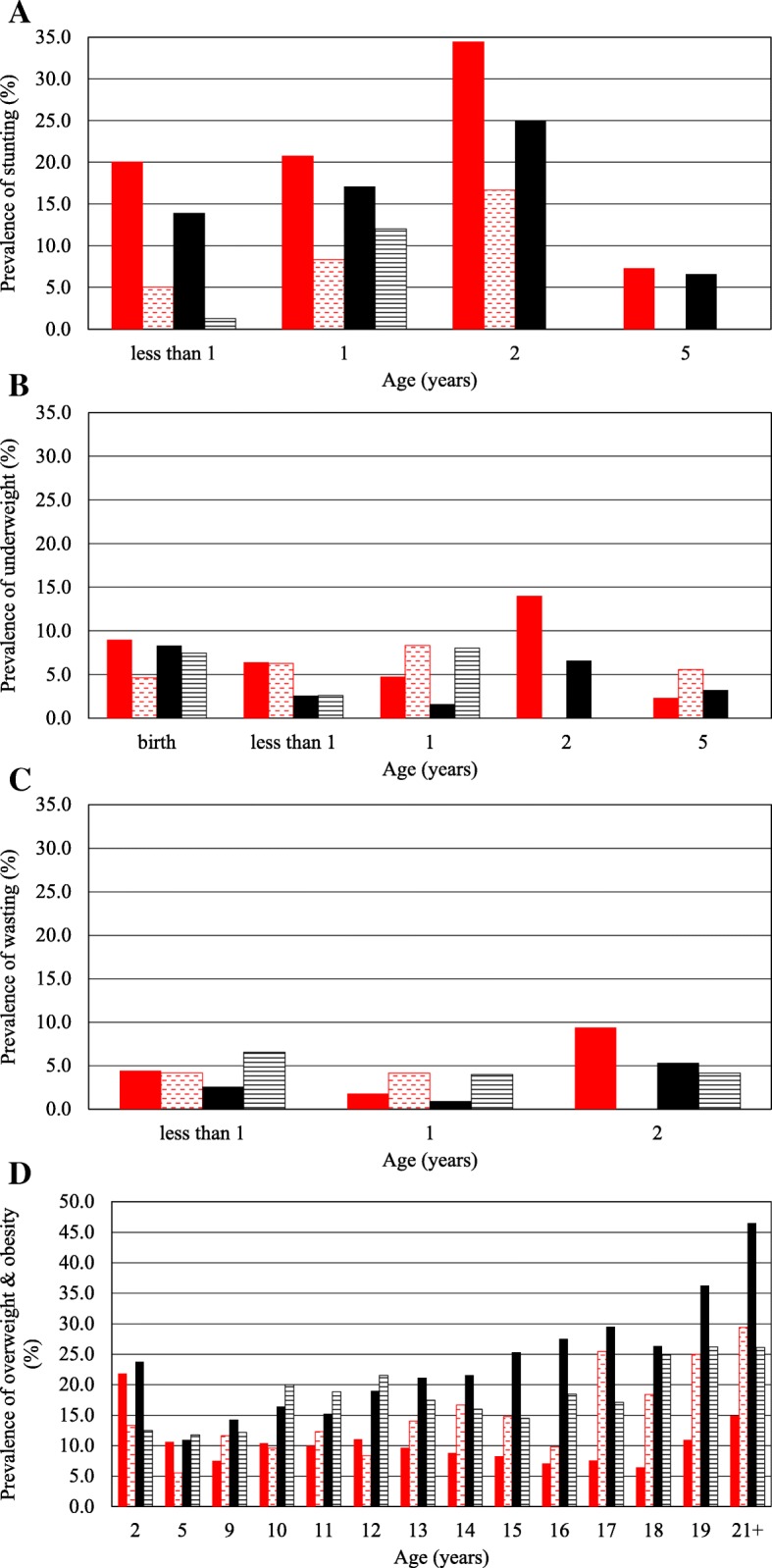
Prevalence of **a** stunting **b** underweight **c** wasting **d** overweight and obesity for black boys (red solid), white boys (red dashed), black girls (black solid), and white girls (black lines)

The prevalence of stunting, underweight and wasting from birth to 5 years, and overweight and obesity from 2 to 21+ years are presented in Fig. 1 and Additional file 1: Table S1. Black children were more stunted than white children at all ages up to and including the 5 year age group, and males were more stunted than females except in the 5 year age group. Stunting reached a peak of 34 and 25% in black males and females at 2 years respectively. Black males tended to be more underweight than black females during the first 3 years of life, while there were no consistent patterns between the race groups in the first 5 years. The prevalence of wasting was low at all times in the first 3 years, although black males in the 2-year age group had a higher prevalence than the other three groups.

In both race and sex groups, the prevalence of overweight and obesity was high in the 2-year age group, but the prevalence then fell in all groups. In black males, the prevalence changed little from age 5 years onwards, remaining below 10% at most time points, reaching a peak of 14.9% at 21+ years. The prevalence in white males was consistently higher than that in black males after 12 years of age, and rose sharply to 29.4% in the 21+ years age group. Females of both race groups had a higher prevalence of overweight and obesity than males throughout childhood and the adolescent period, with black females having a steady rise in prevalence from 11 years of age, reaching 46.5% in the 21+ years age group. The prevalence in white females changed little from 10 years of age, remaining between 15 and 20% throughout adolescence.

### Height, weight, BMI, waist and hip circumference percentiles

**Fig. 2:**
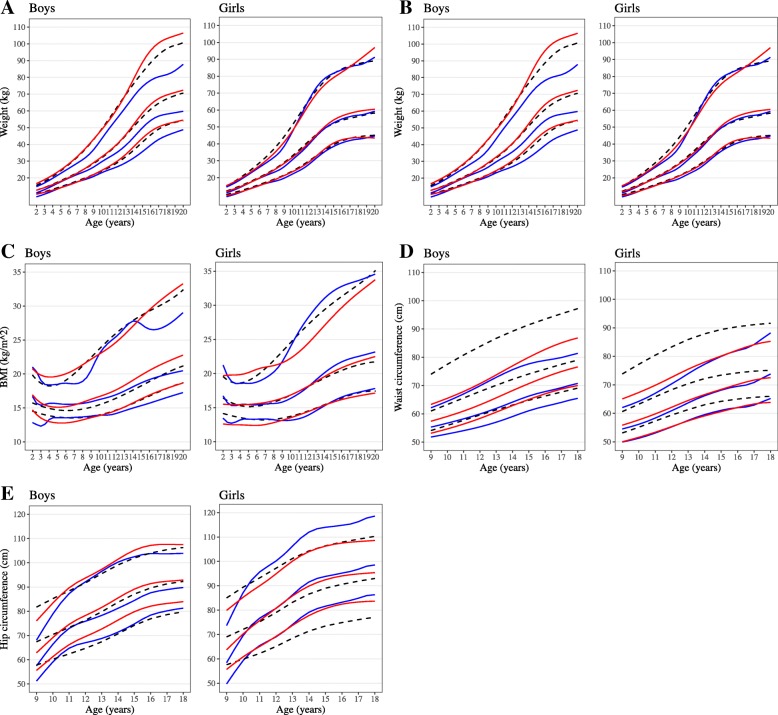
Comparison of centile lines (3, 50, 97 for height, weight & BMI, 10, 50, 90 for waist circumference, and 2.3rd, 50th & 97.7th for hip circumference) between South African black (blue), white (red) children for **a** height, **b** weight, and **c** BMI compared to CDC references (black-dashed), **d** waist circumference compared to NHANES III references (black-dashed), and **e** hip circumference compared to Dutch references (black-dashed)

Values for the 3rd, 50th, and 97th centiles for height, weight and BMI from age 2 to 20 years, 10th, 50th and 90th centiles for waist circumferences and 2.3rd, 50th and 97.7th centiles for hip circumference from 9 to 18 years are given in Table 2. The Bt20+ centile lines for black and white children were superimposed on the 2000 CDC growth charts for height, weight and BMI, on NHANES III reference charts for waist circumference and Dutch references for hip circumferences (Fig. 2). The weight, height and BMI centiles for white Bt20+ children were similar to those of the 2000 CDC charts. However, for black children, height centile lines were lower than those of both the 2000 CDC charts and Bt20+ white children, such that the 50th centile of the black children corresponded to the 25th of the 2000 CDC growth charts for both sexes. A similar pattern was observed for weight and BMI centile lines for black males, but differed for black females, whose lines were similar to the 2000 CDC charts and those of Bt20+ white females.

**Table 2:** Percentile values for the 3rd, 50th, and 97th centiles for (a) height, (b) weight, (c) BMI, (d), 10th, 50th and 90th centiles for waist circumference, and (e) 2.3rd, 50th and 97.7th for hip circumference

(a) Height												
Age (years)	Black males (cm)			White males (cm)			Black females (cm)			White females (cm)		
	C3	C50	C97	C3	C50	C97	C3	C50	C97	C3	C50	C97
2	75.7	82.5	89.0	80.5	87.3	95.7	74.9	81.7	88.5	79.9	86.0	94.4
3	83.9	91.2	98.2	86.8	93.9	102.6	83.3	90.6	97.8	86.6	93.2	102.5
4	91.6	99.4	106.9	93.3	100.6	109.7	91.2	99.0	106.8	93.2	100.5	110.6
5	98.5	106.7	114.7	99.4	107.2	116.7	97.8	106.3	114.7	99.6	107.6	118.4
6	104.5	113.4	122.0	105.1	113.3	123.4	104.1	113.2	122.3	105.6	114.2	125.8
7	109.6	119.2	128.6	110.3	119.1	129.9	109.1	118.8	128.4	111.1	120.3	132.6
8	113.6	123.9	134.2	115.7	125.2	136.8	112.8	122.9	133.0	116.4	126.2	139.2
9	118.3	129.2	140.2	121.9	132.1	144.8	118.2	129.1	139.9	121.9	132.2	145.8
10	123.7	134.9	146.6	128.0	138.9	152.6	124.3	136.2	148.0	127.4	138.3	152.5
11	128.4	140.1	152.5	133.3	145.0	159.8	129.7	142.5	155.1	133.1	144.5	159.1
12	131.9	144.6	158.1	138.6	151.1	167.0	135.5	148.4	161.2	139.0	150.7	165.7
13	135.7	149.9	164.7	144.5	157.7	174.8	141.1	153.4	165.5	144.4	156.4	171.5
14	141.1	156.6	172.1	151.0	164.8	182.7	144.9	156.5	168.0	148.4	160.6	175.7
15	147.3	162.9	177.8	156.9	171.1	189.4	146.6	158.0	169.2	150.9	163.0	177.8
16	152.8	167.3	180.8	160.9	175.1	193.4	147.3	158.7	169.9	152.1	164.2	178.6
17	156.3	169.9	182.2	162.9	176.9	194.9	147.7	159.1	170.5	152.8	164.7	178.8
18	158.0	171.1	183.0	163.7	177.5	195.1	147.9	159.5	170.9	153.2	165.0	178.8
19	158.0	171.1	182.9	164.2	177.7	194.9	147.4	159.0	170.5	153.3	165.0	178.5
20	156.7	169.9	181.9	165.0	178.3	195.0	146.5	158.1	169.6	153.3	164.8	178.0
(b) Weight												
Age (years)	Black males (kg)			White males (kg)			Black females (kg)			White females (kg)		
	C3	C50	C97	C3	C50	C97	C3	C50	C97	C3	C50	C97
2	8.7	11.1	14.8	10.3	12.7	16.6	8.7	11.1	14.8	10.3	12.7	16.6
3	10.3	13.0	17.0	11.4	14.2	19.0	10.3	13.0	17.0	11.4	14.2	19.0
4	12.2	15.3	19.8	12.7	16.0	21.7	12.2	15.3	19.8	12.7	16.0	21.7
5	14.2	17.7	22.8	14.2	18.0	24.8	14.2	17.7	22.8	14.2	18.0	24.8
6	16.0	20.0	25.8	15.8	20.3	28.5	16.0	20.0	25.8	15.8	20.3	28.5
7	17.7	22.0	28.8	17.6	22.8	32.7	17.7	22.0	28.8	17.6	22.8	32.7
8	19.1	24.0	32.1	19.6	25.8	37.6	19.1	24.0	32.1	19.6	25.8	37.6
9	20.9	26.4	36.8	22.0	29.0	43.0	20.9	26.4	36.8	22.0	29.0	43.0
10	23.1	29.6	43.3	24.4	32.5	48.8	23.1	29.6	43.3	24.4	32.5	48.8
11	25.0	32.6	50.0	27.1	36.3	55.0	25.0	32.6	50.0	27.1	36.3	55.0
12	26.8	35.6	56.2	30.3	40.7	62.0	26.8	35.6	56.2	30.3	40.7	62.0
13	29.2	39.6	62.5	34.2	46.1	70.3	29.2	39.6	62.5	34.2	46.1	70.3
14	32.1	44.7	69.1	38.9	52.4	79.8	32.1	44.7	69.1	38.9	52.4	79.8
15	35.7	49.9	74.5	43.7	58.7	88.9	35.7	49.9	74.5	43.7	58.7	88.9
16	39.5	54.2	78.2	47.7	63.9	96.0	39.5	54.2	78.2	47.7	63.9	96.0
17	43.0	56.9	80.4	50.5	67.4	100.6	43.0	56.9	80.4	50.5	67.4	100.6
18	45.3	58.1	81.6	52.2	69.5	103.2	45.3	58.1	81.6	52.2	69.5	103.2
19	47.1	58.9	84.0	53.4	71.0	104.9	47.1	58.9	84.0	53.4	71.0	104.9
20	48.8	59.8	87.8	54.5	72.4	106.5	48.8	59.8	87.8	54.5	72.4	106.5
(c) BMI												
Age (years)	Black males (kg/m^2)			White males (kg/m^2)			Black females (kg/m^2)			White females (kg/m^2)		
	C3	C50	C97	C3	C50	C97	C3	C50	C97	C3	C50	C97
2	12.8	16.7	21.0	14.7	16.9	20.8	13.5	16.7	21.2	12.6	15.5	19.7
3	12.3	15.4	19.1	13.5	15.7	19.8	12.7	15.3	18.8	12.5	15.4	19.8
4	13.3	15.7	18.4	13.0	15.2	19.5	13.2	15.5	18.6	12.5	15.4	19.8
5	13.6	15.6	18.5	12.8	15.1	19.7	13.3	15.4	18.5	12.4	15.5	20.1
6	13.5	15.5	18.6	12.8	15.2	20.0	13.3	15.4	18.8	12.4	15.6	20.6
7	13.6	15.6	18.5	13.0	15.4	20.6	13.3	15.5	19.3	12.5	15.8	20.9
8	13.7	15.7	18.9	13.2	15.8	21.4	13.2	15.6	20.2	12.8	16.0	21.2
9	13.8	15.9	20.5	13.6	16.3	22.2	13.1	15.8	21.7	13.1	16.4	21.6
10	13.9	16.2	23.0	13.9	16.7	22.9	13.2	16.4	24.0	13.5	16.9	22.2
11	13.9	16.5	24.5	14.2	17.1	23.7	13.4	17.1	26.2	13.9	17.4	23.2
12	14.2	16.9	25.5	14.6	17.6	24.7	13.8	18.0	28.1	14.4	18.1	24.4
13	14.6	17.5	26.7	15.1	18.3	25.8	14.5	19.1	29.7	14.9	18.8	25.7
14	15.0	18.1	27.8	15.7	19.1	27.0	15.3	20.1	31.0	15.4	19.5	27.0
15	15.3	18.7	27.3	16.3	19.8	28.3	16.0	21.0	32.1	15.8	20.2	28.4
16	15.7	19.2	26.6	16.9	20.5	29.5	16.5	21.7	32.8	16.2	20.8	29.6
17	16.1	19.6	26.6	17.4	21.2	30.5	16.9	22.2	33.2	16.5	21.3	30.8
18	16.5	19.9	27.1	17.9	21.7	31.5	17.2	22.5	33.6	16.7	21.8	31.8
19	16.9	20.2	27.9	18.3	22.3	32.4	17.5	22.8	34.1	16.9	22.1	32.8
20	17.3	20.5	29.0	18.7	22.8	33.3	17.8	23.2	34.6	17.1	22.5	33.8
(d) Waist circumference												
Age (years)	Black males (cm)			White males (cm)			Black females (cm)			White females (cm)		
	C10	C50	C90	C10	C50	C90	C10	C50	C90	C10	C50	C90
9	51.1	55.4	63.8	51.0	57.1	65.7	48.7	54.5	66.6	50.3	57.2	70.0
10	52.0	56.8	66.4	52.9	59.1	69.0	49.9	56.1	69.0	50.7	57.8	71.1
11	53.0	58.3	69.4	54.8	61.1	72.4	51.6	58.3	72.1	51.9	59.4	73.3
12	54.1	59.9	72.5	56.8	63.3	75.8	53.7	60.9	75.9	53.8	62.0	76.8
13	55.6	61.9	75.6	58.8	65.7	79.3	55.8	63.6	79.9	56.0	64.8	80.5
14	57.5	64.1	78.4	60.9	68.1	83.2	57.8	66.2	83.7	57.4	66.7	83.2
15	59.4	66.3	80.2	63.0	70.4	87.3	59.2	68.2	86.7	58.1	67.9	85.2
16	60.9	67.9	81.2	65.0	72.8	90.8	60.1	69.6	89.1	59.5	70.0	88.1
17	62.2	69.2	81.7	66.7	75.1	92.7	60.7	70.8	91.2	61.3	72.5	91.6
18	63.7	70.9	83.0	67.9	77.4	92.9	63.1	73.9	95.9	61.6	73.2	92.9
19	64.2	71.5	83.4	68.2	79.7	92.9	61.5	72.5	94.7	59.5	71.3	90.8
20	64.2	71.8	83.6	66.4	82.0	93.4	57.8	68.7	90.3	56.6	68.3	87.4
(e) Hip circumference												
Age (years)	Black males (cm)			White males (cm)			Black females (cm)			White females (cm)		
	C2.3	C50	C97.7	C2.3	C50	C97.7	C2.3	C50	C97.7	C2.3	C50	C97.7
9	51.3	57.2	67.4	56.3	63.3	76.1	49.7	57.7	70.4	55.7	63.9	80.1
10	59.5	66.4	78.3	61.5	69.1	82.6	60.1	69.7	85.0	61.0	70.3	85.2
11	65.1	72.9	86.4	66.5	74.5	88.6	65.9	76.6	94.0	65.2	75.7	89.6
12	67.4	75.9	91.0	69.9	78.2	92.6	69.4	80.7	99.2	69.5	80.8	94.4
13	69.2	78.3	95.0	73.0	81.5	96.0	74.5	86.3	105.4	74.1	86.0	99.6
14	71.8	81.2	98.2	76.6	85.4	100.2	79.5	91.6	110.9	78.1	90.0	103.7
15	75.2	84.5	100.4	80.2	89.2	104.2	82.0	94.0	113.0	80.9	92.6	106.0
16	78.7	87.6	102.0	82.5	91.6	106.5	83.5	95.2	113.4	82.7	94.0	107.3
17	80.3	88.9	102.3	83.5	92.5	107.2	85.1	96.7	114.7	83.7	94.7	107.8
18	81.3	89.9	103.0	84.0	92.9	107.3	86.6	98.7	117.7	84.3	95.1	107.9
19	80.1	88.6	101.5	85.2	94.1	108.2	84.5	97.0	117.5	84.8	95.3	108.0
20	78.2	86.6	99.3	86.9	95.8	109.8	81.1	94.0	116.4	85.2	95.5	108.1

The centile lines for waist circumferences for Bt20+ children of both sexes were lower than the NHANES III. During adolescence, the 90th centile of Bt20+ black and white males was just above the 50th centile of the American males. While the 50th centile of Bt20+ white males was below the NHANES III males, the 10th centile for Bt20+ white and NHANES III males were similar. Bt20+ black and white females showed similar patterns and were lower than the NHANES III females. The bands of NHANES III appeared wider than those of the Bt20+ sample. For hip circumference, centile lines for black children showed an acceleration between 9 and 11 years from lower centiles approaching centiles of white children between 10 and 11 years. The 2.3rd centile of Bt20+ black and white females were higher than that of Dutch females. Bt20+ black and white girls showed similar patterns except for the 97.7th centile, which were higher in black than white females.

## Discussion

We assessed the prevalence of malnutrition among a population of urban South African children and generated percentile values for children living in the Johannesburg metropolitan region. Between birth and 5 years, the prevalence of stunting was higher in black than white children and in males than females reaching a peak at 2 years of age at over 34% for black males. This is higher than the national average of 26% for children from birth to 3 years [33] and remarkably higher than the 6% average for children from birth to 5 years from HIC [4]. Micronutrient deficiency in early childhood may be responsible for the high rate of undernutrition in black children [33]. On the contrary, the prevalence of overweight and obesity of South African females were comparable to those of American females who had an average of 26.5 and 41.9% for non-Hispanic white and African American females respectively between 12 and 19 years of age [34]. In our sample, the prevalence of overweight and obesity ranged from about 19 to 36% and from about 22 to 26% between 12 and 19 years of age for black and white females respectively. In adulthood, black women have a higher prevalence of obesity than white women while the prevalence of overweight is similar between the two groups [35].

The rise in the prevalence of overweight and obesity may be attributed to poor dietary patterns and low levels of physical activity among black children. Black children of both sexes in the Bt20+ cohort had significantly lower levels of reported physical activity than white children [36]. Additionally, among Bt20+ black children, dietary patterns differed with a greater proportion of females consuming snacks and confectionary and a lower proportion taking lunchboxes to school than males, contributing to possibly higher consumption of fats and refined sugar [37]. Residential mobility among those experiencing an increase in SES between birth and 15 years of age was associated with a greater BMI in females, but not in males. Thus, the socio-economic transition may predispose females to greater exposure to bad dietary practices both within and without the household, while maintaining similarly low levels of physical activity compared to males.

Changing dietary patterns could be driving a positive secular change in BMI such that the centiles for black females are now similar to those of white South African females and American references. Historically, black females had lower weight and adiposity than white females [7, 9]. Compared to two nationally representative references of American children, the weight and BMI centiles for Bt20+ females are similar to American references for all centiles. There are no historical growth reference data to assess whether the secular change in BMI is affecting all centiles and thus it is unclear whether in South Africa, the rise in BMI affects those in a select group or affect all centiles. In America, the rise in obesity was driven by the heaviest sub-group (> 95th centile) being much heavier than the same group in previous surveys, while the lower centiles were similar for children and adolescents between surveys, leading to a widening of the upper centiles [14]. For waist circumference, South African females started lower but grew towards the American references in adolescence. This suggests that the pattern of obesity of younger South African females differs from that of American peers, with general rather than abdominal obesity among South African children.

The percentiles produced in this study will be useful for future comparisons to assess patterns in the secular changes in weight and BMI so as to give insight into factors that drive the obesity epidemic. Nevertheless, this study has several limitations in that the data are not nationally representative and external validity of the Bt20+ sample was only conducted at birth. There were no differences in maternal age, gravidity and birth weight between Bt20+ participants and those born in the same year but outside of the 7 week recruitment period for the cohort [17]. The Soweto-Johannesburg Metropolitan area has experienced significant inward migration and children who recently migrated into this area are significantly more disadvantaged compared to long-term residents [38], thus these percentiles are representative of children born in Soweto in the early 90’s who have had long-term residence in the township. A second limitation of the study is the relatively small number of white children, particularly in early life, even though the proportions represent the demographics of population at the time of birth.

## Conclusions

In conclusion, the availability of data describing growth patterns is a critical tool for current and future comparisons and is essential for public health. More studies are needed to establish whether the positive secular change in weight and BMI affects the general population or a sub-group. This would assist with developing a targeted intervention against obesity. The risk for overweight and obesity in South African females increases in early adolescence, and may be linked to early life growth and physiological and lifestyle changes during that period. Early intervention is needed in females to mitigate against the upsurge of overweight and obesity.

## Additional file

Additional file 1: **Table S1.** Ethnic and sex differences in the prevalence of (a) stunting, (b) underweight, (c) wasting, and (d) overweight and obesity. Sex differences are indicated by asterisks. (DOCX 29 kb)

## References

[1] Kimani-Murage EW, Kahn K, Pettifor JM, Tollman SM, Dunger DB, Gómez-Olivé XF (2010). The prevalence of stunting, overweight and obesity, and metabolic disease risk in rural south African children. BMC Public Health.

[2] de Onis M, Blössner M, Borghi E (2010). Global prevalence and trends of overweight and obesity among preschool children. Am J Clin Nutr.

[3] WHO. Obesity and overweight: WHO; 2017. http://www.who.int/mediacentre/factsheets/fs311/en/. Accessed 8 Dec 2017

[4] de Onis M, Blössner M, Borghi E (2011). Prevalence and trends of stunting among pre-school children: 1990-2020. Public Health Nutr.

[5] Bourne LT, Lambert EV, Steyn K (2002). Where does the black population of South Africa stand on the nutrition transition?. Public Health Nutr.

[6] Steyn NP, Nel JH, Parker W, Ayah R, Mbithe D (2012). Urbanisation and the nutrition transition: a comparison of diet and weight status of south African and Kenyan women. Scand J Public Health.

[7] Kark SL, Le Riche HA (1944). Health study of south African bantu school-children. S Afr Med J.

[8] Chaning-Pearce SM, Solomon L (1986). A longitudinal study of height and weight in black and white Johannesburg children. S Afr Med J.

[9] Smit P (1971). Anthropometric status of Pretoria children of four populations: increases in cross-sectional samples. S Afr Med J.

[10] Cameron N, Kgamphe JS, Leschner KF, Farrant PJ (1992). Urban-rural differences in the growth of south African black children. Ann Hum Biol.

[11] Leary PM, Obst D (1969). The use of percentile charts in the nutritional assessment of children from primitive communities. S Afr Med J.

[12] Cameron N, Gordon-Larsen P, Wrchota EM (1994). Longitudinal analysis of adolescent growth in height, fatness, and fat patterning in rural south African black children. Am J Phys Anthropol.

[13] Cameron N, De Wet T, Ellison GT, Bogin B (1998). Growth in height and weight of south African urban infants from birth to five years: the birth to ten study. Am J Hum Biol.

[14] Flegal KM, Troiano RP (2000). Changes in the distribution of body mass index of adults and children in the US population. Int J Obes.

[15] Pernegger L, Godehart S. Townships in the south African geographic landscape – physical and social legacies and challenges. 2007. http://www.treasury.gov.za/divisions/bo/ndp/TTRI/TTRI%20Oct%202007/Day%201%20-%2029%20Oct%202007/1a%20Keynote%20Address%20Li%20Pernegger%20Paper.pdf. Accessed 13 Dec 2017.

[16] South African History Online. Soweto. 2018. http://www.sahistory.org.za/places/soweto. Accessed 9 Dec 2017.

[17] Richter LM, Yach D, Cameron N, Griesel RD, Wet T (1995). Enrolment into birth to ten (BTT): population and sample characteristics. Paediatr Perinat Epidemiol.

[18] Richter LM, Norris SA, De Wet T (2004). Transition from birth to ten to birth to twenty: the south African cohort reaches 13 years of age. Paediatr Perinat Epidemiol.

[19] Richter L, Norris S, Pettifor J, Yach D, Cameron N (2007). Cohort profile: Mandela’s children: the 1990 birth to twenty study in South Africa. Int J Epidemiol.

[20] May A, Hazelhurst S, Li Y, Norris SA, Govind N, Tikly M (2013). Genetic diversity in black south Africans from Soweto. BMC Genomics.

[21] Cameron N (1984). The measurement of human growth.

[22] Martorell R, Lohman TG, Roche AF (1991). Anthropometric standardization reference manual.

[23] Cole TJ (1990). The LMS method for constructing normalized growth standards. Eur J Clin Nutr.

[24] Stasinopoulos DM, Rigby RA (2007). Generalized additive models for location scale and shape (GAMLSS) in R. J Stat Softw.

[25] Kuczmarski RJ, Ogden CL, Guo SS, Grummer-Strawn LM, Flegal KM, Mei Z (2002). 2000 CDC growth charts for the United States: methods and development. Vital Health Stat.

[26] Cook S, Auinger P, T-K Huang T (2009). Growth curves for cardio-metabolic risk factors in children and adolescents. J Pediatr.

[27] Fredriks AM, Van Buuren S, Fekkes M, Verloove-Vanhorick SP, Wit JM (2005). Are age references for waist circumference, hip circumference and waist-hip ratio in Dutch children useful in clinical practice?. Eur J Pediatr.

[28] de Onis M, Garza C, Onyango AW, Rolland-Cachera MF (2009). WHO growth standards for infants and young children. Arch Pediatr.

[29] Cole TJ, Bellizzi MC, Flegal KM, Dietz WH (2000). Establishing a standard definition for child overweight and obesity worldwide: international survey. BMJ.

[30] WHO. Application tools: WHO; 2013. http://www.who.int/growthref/tools/en/. Accessed 12 Dec 2017

[31] WHO. Child growth standards: WHO; 2017. http://www.who.int/childgrowth/software/en/. Accessed 12 Dec 2017.

[32] Cole TJ, Donaldson MDC, Ben-Shlomo Y (2010). SITAR--a useful instrument for growth curve analysis. Int J Epidemiol.

[33] Shisana O, Labadarios D, Rehle T, Simbayi L, Zuma K, Dhansay A (2013). South African National Health and nutrition examination survey.

[34] Hedley AA, Ogden CL, Johnson CL, Carroll MD, Curtin LR, Flegal KM (2004). Prevalence of overweight and obesity among US children, adolescents, and adults, 1999-2002. JAMA.

[35] Puoane T, Steyn K, Bradshaw D, Laubscher R, Fourie J, Lambert V (2002). Obesity in South Africa: the south African demographic and health survey. Obes Res.

[36] McVeigh JA (2004). The relationship between socio-economic status and physical activity patterns in south African children. Acta Paediatr.

[37] Feeley AB, Musenge E, Pettifor JM, Norris SA (2012). Investigation into longitudinal dietary behaviours and household socio-economic indicators and their association with BMI Z-score and fat mass in south African adolescents: the birth to twenty (Bt20) cohort. Public Health Nutr.

[38] Richter LM, Norris SA, Swart TM, Ginsburg C (2006). In-migration and living conditions of young adolescents in greater Johannesburg, South Africa. Soc Dyn.

