# Supplementary material for: The prevalence of malnutrition and growth percentiles for urban South African children
Source: BMC Public Health. 2019 May 2;19:492. doi: 10.1186/s12889-019-6794-1 (PMC6498578; doi:10.1186/s12889-019-6794-1)
Supplement: Supplementary file 1 — Table S1. Ethnic and sex differences in the prevalence of (a) stunting, (b) underweight, (c) wasting, and (d) overweight and obesity. Sex differences are indicated by asterisks. (DOCX 29 kb) [file 12889_2019_6794_MOESM1_ESM.docx]

**Table S1: Ethnic and sex differences in the prevalence of (a) stunting, (b) underweight, (c) wasting, and (d) overweight and obesity. Sex differences are indicated by asterisks**

1. **Stunting**

| Age group | Black boys | | White boys | | P-value | Black girls | | White girls | | P-value |
| --- | --- | --- | --- | --- | --- | --- | --- | --- | --- | --- |
|  | n | freq (%) | n | freq (%) |  | n | freq (%) | n | freq (%) |  |
| < 1 | 588 | 118 (20.1)** | 99 | 5 (5.1) | **<0.0001** | 582 | 81 (13.9) | 79 | 1 (1.3) | **0.001** |
| 1 | 423 | 88 (20.8) | 24 | 2 (8.3) | 0.138 | 467 | 80 (17.1) | 25 | 3 (12.0) | 0.505 |
| 2 | 299 | 103 (34.4)* | 30 | 5 (16.7)* | **0.048** | 305 | 76 (24.9) | 24 | 0 (0.0) | **<0.0001** |
| 5 | 519 | 38 (7.3) | 18 | 0 (0.0) | 0.234 | 561 | 37 (6.6) | 17 | 0 (0.0) | 0.274 |

1. **Underweight**

| Age group | Black boys | | White boys | | | P-value | Black girls | | White girls | | P-value |
| --- | --- | --- | --- | --- | --- | --- | --- | --- | --- | --- | --- |
|  | n | freq (%) | n | freq (%) |  | | n | freq (%) | n | freq (%) |  |
| birth | 1245 | 112 (9.0) | 152 | 7 (4.6) | 0.067 | | 1324 | 110 (8.3) | 148 | 11 (7.4) | 0.713 |
| < 1 | 591 | 38 (6.4)** | 96 | 6 (6.3) | 0.947 | | 580 | 15 (2.6) | 77 | 2 (2.6) | 0.995 |
| 1 | 402 | 19 (4.7)** | 24 | 2 (8.3) | 0.428 | | 441 | 7 (1.6) | 25 | 2 (8.0) | **0.023** |
| 2 | 300 | 42 (14.0)** | 31 | 0 (0.0) | **0.026** | | 304 | 20 (6.6) | 24 | 0 (0.0) | 0.195 |
| 5 | 519 | 12 (2.3) | 18 | 1 (5.6) | 0.379 | | 561 | 18 (3.2) | 17 | 0 (0.0) | 0.532 |

1. **Wasting**

| Age group | Black boys | | White boys | | P-value | Black girls | | White girls | | P-value |
| --- | --- | --- | --- | --- | --- | --- | --- | --- | --- | --- |
|  | n | freq (%) | n | freq (%) |  | n | freq (%) | n | freq (%) |  |
| < 1 | 588 | 26 (4.4) | 95 | 4 (4.2) | 0.926 | 580 | 15 (2.6) | 76 | 5 (6.6) | 0.057 |
| 1 | 398 | 7 (1.8) | 24 | 1 (4.2) | 0.401 | 439 | 4 (0.9) | 25 | 1 (4.0) | 0.146 |
| 2 | 299 | 28 (9.4) | 30 | 0 (0.0) | **0.030** | 303 | 16 (5.3) | 24 | 1 (4.2) | 0.813 |

1. **Overweight & obesity**

| Age  group | Black boys | | White boys | | P-value | Black girls | | White girls | | P-value |
| --- | --- | --- | --- | --- | --- | --- | --- | --- | --- | --- |
|  | n | freq (%) | n | freq (%) |  | n | freq (%) | n | freq (%) |  |
| 2 | 298 | 65 (21.8) | 30 | 4 (13.3) | 0.277 | 299 | 71 (23.7) | 24 | 3 (12.5) | 0.207 |
| 5 | 519 | 55 (10.6) | 18 | 1 (5.6) | 0.491 | 560 | 61 (10.9) | 17 | 2 (11.8) | 0.901 |
| 9 | 255 | 19 (7.5)* | 43 | 5 (11.6) | 0.352 | 253 | 36 (14.2) | 41 | 5 (12.2) | 0.727 |
| 10 | 183 | 19 (10.4) | 73 | 7 (9.6) | 0.850 | 159 | 26 (16.4) | 65 | 13 (20.0) | 0.513 |
| 11 | 472 | 47 (10.0)* | 73 | 9 (12.3) | 0.535 | 506 | 77 (15.2) | 69 | 13 (18.8) | 0.437 |
| 12 | 616 | 68 (11.0)*** | 72 | 6 (8.3)* | 0.483 | 702 | 133 (18.9) | 79 | 17 (21.5) | 0.582 |
| 13 | 643 | 62 (9.6)*** | 57 | 8 (14.0) | 0.289 | 726 | 153 (21.1) | 63 | 11 (17.5) | 0.498 |
| 14 | 767 | 67 (8.7)*** | 60 | 10 (16.7) | 0.042 | 809 | 174 (21.5) | 75 | 12 (16.0) | 0.263 |
| 15 | 803 | 66 (8.2)*** | 61 | 9 (14.8) | 0.081 | 886 | 224 (25.3) | 62 | 9 (14.5) | 0.057 |
| 16 | 722 | 51 (7.1)*** | 61 | 6 (9.8) | 0.424 | 749 | 206 (27.5) | 65 | 12 (18.5) | 0.114 |
| 17 | 581 | 44 (7.6)*** | 55 | 14 (25.5) | **0.000** | 645 | 190 (29.5) | 70 | 12 (17.1) | **0.030** |
| 18 | 452 | 29 (6.4)*** | 38 | 7 (18.4) | **0.016** | 433 | 114 (26.3) | 36 | 9 (25.0) | 1.000 |
| 19 | 55 | 6 (10.9)** | 40 | 10 (25.0) | 0.125 | 47 | 17 (36.2) | 42 | 11 (26.2) | 0.433 |
| 21+ | 756 | 113 (14.9)*** | 17 | 5 (29.4) | 0.194 | 748 | 348 (46.5) | 23 | 6 (26.1) | 0.085 |

Age group < 1 = age between 3 months and 1 year; *p < 0.05; **p < 0.01, ***p < 0.001
